# Paving the way for the production of secretory proteins by yeast cell factories

**DOI:** 10.1111/1751-7915.13342

**Published:** 2019-02-08

**Authors:** Maria José Huertas, Carmen Michán

**Affiliations:** ^1^ Instituto de Bioquímica Vegetal y Fotosíntesis Universidad de Sevilla‐CSIC Américo Vespucio 49 41092 Sevilla Spain; ^2^ Departamento de Bioquímica y Biología Molecular Campus de Excelencia Internacional Agroalimentario CeiA3 Universidad de Córdoba Edificio Severo Ochoa C‐6, 2^a^Planta 14071 Córdoba Spain

Humans have used yeasts for the production of food and beverages since ancient times. Currently, *Saccharomyces cerevisiae* is also a key platform for the production of molecules with biopharmaceutical and industrial relevance. Yeasts are excellent choices as cell factories for recombinant proteins due to several reasons, including: (i) rapid growth, (ii) easy genetic manipulation, (iii) complete genome sequences usually available, plus (iv) their eukayotic machinery for post‐transcriptional and post‐translational modifications. But yeasts are much more than *S. cerevisiae*. The ‘other’ yeasts, so‐called ‘non‐conventional yeasts’, include a wide spectrum of species, barely exploited, with multiple peculiar physiological and molecular characteristics. Lately, methylotrophic yeast *Pichia pastoris* (*a.k.a Komagataella phaffi* or *K. pastori*) stands out as the ideal host for heterologous expression, basically due to three properties: its capacity to grow until very high densities, its ability to secrete fully proper folded and functional proteins, and its *AOX1* (Alcohol oxidase I) promoter, that is both strongly repressed in the presence of glucose, glycerol or ethanol, and fully induced by methanol (Ahmad *et al*., 2014; Looser *et al*., 2015; Yang *et al*., 2018). Nevertheless, using non‐conventional yeasts for biotechnological applications usually clashes with a main bottleneck, as the process for cloning the desired genes in not an easy and direct procedure but sometimes a long and winding road.

Furthermore, in the last years a large amount of data about previously unknown genes or proteins has been generated by the ‘omics’ techniques. A significant step to study the biological functions of these proteins is its purification what needs a suitable system for their heterologous expression. This hindrance has been addressed in a recent report for Microbial Biotechnology where González and coworkers describe a tool that allows the high‐throughput expression of proteins in *S. cerevisiae* and *P. pastoris*. in a simple and fast way. The protocol involves the expression of heterologous proteins by transformation of *S. cerevisiae* with a PCR product that carries the gene of interest, obtained from the original cDNA, and its integration either into the *S. cerevisiae* genome or in an autonomous replicative plasmid (pYEDIS) that contains the *P. pastoris AOX1* promoter. In the last procedure, a cloning step can be avoided as the genetic engineered plasmid is generated by homologous recombination between two fragments that are co‐transformed into the yeast cell: one with the PCR product, and the other with a linearized vector containing different elements that drive expression and secretion of the recombinant protein. This plasmid can then be isolated from *S. cerevisiae* and directly used to transform *P. pastoris* to express the target proteins, as pYEDIS vector includes the elements necessary for expression in *P. pastoris*. Also, this tool could be used for other applications as expression of intracellular protein or chimeric proteins, only requiring PCR reactions with the corresponding primers, and a one step transformation in *S. cerevisiae*. However, to take full advantage of these cell factories, other limitations constraining product yields need to be addressed. Further major restrictions are related to the differences in post‐translational modifications between yeasts and other eukaryotes, and in the extra effort required for the production of a high amount of protein products. These processes encompass multiple steps and thousands of genes but their imbalance usually leads to the induction of the unfolded protein response and to protein degradation which deeply affects the system efficiency (de Ruijter *et al*., 2016a,b). Several strategies are currently being used to overcome protein folding limitations. In *P. pastoris*, overexpressing genes involved in protein folding have been partially successful*,* both with exogenous (*Escherichia coli gro* chaperones) or endogenous genes (*PDI1*,* ERO1* and *GPX1*) (Ben Azoun *et al*., 2016; Summpunn *et al*., 2018). Furthermore, in *S. cerevisiae*, IgGs secretion yields have been additionally improved when the overexpression of those genes was combined with an increment in the endoplasmic reticulum size (de Ruijter *et al*., 2016a,b).

General shortcomings related to the industrial production steps also need to be considered, including multi‐tolerance towards high temperature, extreme pH, shearing forces, ethanol or inhibitory compounds found in alternative cheaper carbon sources. Two non‐excluding approaches can be followed: the evaluation of ‘environmental’ isolates, or the targeted modification of laboratory strains. Both ways are being used for the improvement of heterologous cellulase secretion in *S. cerevisiae*: several environmental‐isolated strains have been evaluated identifying excellent candidates for industrial purposes (Davison *et al*., 2016), and also a laboratory strain has been modified with an engineered improved vesicle trafficking mechanism (Tang *et al*., 2017). Besides, *AOX1* promoter is commonly used in *P. pastoris* constructions but has also its limitations. Methanol is highly flammable so its use as an effector for the promoter is not convenient in large industrial facilities. This restriction can be overcome either by using other promoters (*GAP*,* TEF*,* PGK*,* ADH1*,* ENO1*, etc.) or by modifying *AOX1* (changing its transcription factors binding sites, dA:dT tracts, etc.), and both strategies are also currently under investigation (Ahmad *et al*., 2014; Yang *et al*., 2018).

We must not lose sight that other ‘less known’ yeasts could also be good candidates as hosts for heterologous secretory expression as thermotolerant *Kluyveromyces marxianus* (Gombert *et al*., 2016) or halotolerant *Debaryomyces hansenii* (Prista *et al*., 2016), although they face similar problems as the ones described above.

Finally, some of the existing editing problems could be addressed by a synthetic biology approach using new tools as the CRISP/Cas9 system or novel projects as *de novo* synthesizing yeast chromosomes. Nevertheless, there is still a long road ahead as new genetic constructions of non‐conventional yeast strains for biotechnological applications are still far from achieving the maturity of *S. cerevisiae* systems. Only an integrating approach from multiple perspectives will allow the achievement of top yields in the production of secretory proteins for a satisfactory industrial applicability of the system.

## Conflict of interest

None declared.

## References

[mbt213342-bib-0001] Ahmad, M. , Hirz, M. , Pichler, H. , and Schwab, H. (2014) Protein expression in *Pichia pastoris*: recent achievements and perspectives for heterologous protein production. Appl Microbiol Biotechnol 98: 5301–5317. 24743983 10.1007/s00253-014-5732-5 PMC4047484

[mbt213342-bib-0002] Ben Azoun, S. , Belhaj, A.E. , Gongrich, R. , Gasser, B. , and Kallel, H. (2016) Molecular optimization of rabies virus glycoprotein expression in *Pichia pastoris* . Microb Biotechnol 9: 355–368. 26880068 10.1111/1751-7915.12350 PMC4835572

[mbt213342-bib-0003] Davison, S.A. , den Haan, R. , and van Zyl, W.H. (2016) Heterologous expression of cellulase genes in natural *Saccharomyces cerevisiae* strains. Appl Microbiol Biotechnol 100: 8241–8254. 27470141 10.1007/s00253-016-7735-x

[mbt213342-bib-0004] de Ruijter, J.C. , Jurgens, G. and Frey, A.D. (2016a) Screening for novel genes of Saccharomyces cerevisiae involved in recombinant antibody production. FEMS Yeast Res 17, fow104. 27956492 10.1093/femsyr/fow104

[mbt213342-bib-0005] de Ruijter, J.C. , Koskela, E.V. , and Frey, A.D. (2016b) Enhancing antibody folding and secretion by tailoring the Saccharomyces cerevisiae endoplasmic reticulum. Microb Cell Fact 15: 87. 27216259 10.1186/s12934-016-0488-5 PMC4878073

[mbt213342-bib-0006] Gombert, A.K. , Madeira, J.V. Jr , Cerdan, M.E. , and Gonzalez‐Siso, M.I. (2016) *Kluyveromyces marxianus* as a host for heterologous protein synthesis. Appl Microbiol Biotechnol 100: 6193–6208. 27260286 10.1007/s00253-016-7645-y

[mbt213342-bib-0007] Looser, V. , Bruhlmann, B. , Bumbak, F. , Stenger, C. , Costa, M. , Camattari, A. , *et al* (2015) Cultivation strategies to enhance productivity of *Pichia pastoris*: a review. Biotechnol Adv 33: 1177–1193. 26027890 10.1016/j.biotechadv.2015.05.008

[mbt213342-bib-0008] Prista, C. , Michan, C. , Miranda, I.M. , and Ramos, J. (2016) The halotolerant *Debaryomyces hansenii*, the Cinderella of non‐conventional yeasts. Yeast (Chichester, England) 33: 523–533. 10.1002/yea.3177 27279567

[mbt213342-bib-0009] Summpunn, P. , Jomrit, J. , and Panbangred, W. (2018) Improvement of extracellular bacterial protein production in *Pichia pastoris* by co‐expression of endoplasmic reticulum residing GroEL‐GroES. J Biosci Bioeng 125: 268–274. 29046263 10.1016/j.jbiosc.2017.09.007

[mbt213342-bib-0010] Tang, H. , Song, M. , He, Y. , Wang, J. , Wang, S. , Shen, Y. , *et al* (2017) Engineering vesicle trafficking improves the extracellular activity and surface display efficiency of cellulases in *Saccharomyces cerevisiae* . Biotechnol Biofuels 10: 53. 28261326 10.1186/s13068-017-0738-8 PMC5327580

[mbt213342-bib-0011] Yang, J. , Cai, H. , Liu, J. , Zeng, M. , Chen, J. , Cheng, Q. , and Zhang, L. (2018) Controlling *AOX1* promoter strength in *Pichia pastoris* by manipulating poly (dA:dT) tracts. Sci Rep 8: 1401. 29362428 10.1038/s41598-018-19831-y PMC5780452

